# Female Tenacity of Life

**Published:** 1884-07

**Authors:** 


### FEMALE TENACITY OF LIFE.

IT appears from the gathered statistics of the world that women have a greater tenacity of life than men. Nature worships the female in all its varieties. Among insects the male perishes at a relatively earlier period. In plants the seminate blossoms die earliest and are produced on the weaker limbs. Female quadrupeds have more endurance than males. In the human race, despite the intellectual and physical strength of the man, the woman endures longest, and will bear pain to which the strong man succumbs. Zymotic diseases are more fatal to males, and more male children die than females. Deverga asserts that the proportion dying suddenly is about 100 women to 780 men; 1,080 men in the United States in 1870 committed suicide, to 285 women.

Intemperance, apoplexy, gout, hydrocephalus, affections of the heart and liver, scrofula, and paralysis, are far more fatal to males than females. Pulmonary consumption, on the other other hand, is more deadly to the latter. Females in cities are more prone to consumption than in the country. All old countries not disturbed by emigration have a great majority of females in the population. In royal families the statistics show more daughters than sons. The Hebrew woman is exceptionally short lived. The married state is favorable to prolongation of life among woman. Dr. Hough proclaims that there are from 2 to 6 per cent. more males born than females, and yet there are more than 56 per cent. of females in the living population. From which statistics we conclude that all women ought to marry, and that as men are likely to become so scarce they cannot be sufficiently prized by the other sex.

